# Promotion of Values Education (Factors Involved in Prosocial Behaviors and Volunteering)

**DOI:** 10.3390/ejihpe14020028

**Published:** 2024-02-19

**Authors:** María del Carmen Olmos-Gómez, Raquel Portillo-Sánchez, Laila Mohamed-Mohand, Ligia Isabel Estrada-Vidal

**Affiliations:** 1 Department of Research Methods and Diagnosis in Education, Faculty of Education and Sport Sciences, University of Granada, 18071 Granada, Spain; raquelps92@gmail.com (R.P.-S.); ligia@ugr.es (L.I.E.-V.); 2 Department of Developmental and Educational Psychology, Faculty of Education and Sport Sciences, University of Granada, 18071 Granada, Spain

**Keywords:** prosocial, culture, affection, sociability, volunteering, self-transcendence, education, values

## Abstract

(1) Background: Prosocial behavior aligns with the current societal model, where human values hold greater importance considering cultural, social, and personal variables that may influence the opportunity to benefit others. Hence, the objective of this research was established: to understand how diverse factors influence the values of young people, aiming to promote education and enhance prosocial behavior. (2) Methods: This study is quantitative research employing an empirical–analytical, cross-sectional social research method. A validated instrument was used with a sample of 1702 individuals from the city of Melilla, noteworthy for its multicultural context due to its location in North Africa. (3) Results: Inferential analysis was conducted using multiple linear regression to predict future behaviors, focusing on the factors influencing values. Various models were employed, incorporating twelve variables and four scales: sociability, transcendence, culture, and effects. (4) Conclusions: The results and conclusions suggest the need to enhance affect and sociability, primarily among the most prominent factors.

## 1. Introduction

All human behaviors are related to weighing the cost–benefit balance of actions taken. Prosocial behaviors become an exception to this norm, as they seek to benefit other individuals, where the benefit is perceived as the received gratitude. A key characteristic is fulfilling personal needs by performing diverse tasks, referred to as prosocial values—understood as the opportunity to benefit others. During these life stages, human exploration in different life areas is fundamental for testing and selecting a life plan related to work, lifestyle, and interpersonal relationships [1].

Hamilton and Adamson [2] understood prosocial behaviors as actions carried out by an individual with a clear intention to help and benefit another person or group. This includes acts of aiding, collaborating, caring, donating, and sharing, always with the recipient’s well-being in mind. Such capability can be considered highly beneficial both for the individual personally and for society as a whole [3,4]. Educational programs must focus on continuously and permanently teaching good habits, active citizenship, and responsibility to shape critical citizens. 

When an individual is aware that mental strength is focused on themselves, they become conscious of their thoughts and the needs of others [5]. Rubalcaba-Romero et al. [6] demonstrate the predictive value of socio-emotional skills in prosocial behavior.

Several articles provide evidence that mindfulness, exhibited by a consciously aware person, is considered a factor related to internal motivation in the prosocial domain. It maintains an indirect relationship with prosocial behavior through social attention. In essence, this motivation, attention, and behavior assist individuals in effectively translating intrinsic values and motives [7,8].

The theory of social learning [9] asserts that humans have a unique capacity to fulfill multiple functions. The development of quality and available ways of life is partially determined by an individual’s autonomy and the cultural institutions shaping their overall development. Human development is a heterogeneous phenomenon involving different abilities, following different paths of change, and subject to changes throughout existence. Social systems that provide crosscutting and generalizing capabilities create structures and facilitators, offer supportive resources, leave room for individual autonomy, and increase opportunities for everyone to fulfill their aspirations.

In an increasingly diverse society, it becomes crucial to grasp the inherent values within various cultures. This understanding is essential as conflicts arise in coexistence when individuals from one religious background impose their norms on those from another, as highlighted by Somaraju [10]. Culture, in an ethnographic sense, is a complex whole that encompasses knowledge, beliefs, arts, morality, laws, customs, and all other abilities and habits acquired by an individual as a member of society [10].

Transcendence is understood as benevolence and consideration for a greater good that acknowledges impacts on other individuals, society, and the environment, with its primary objective being the creation of social value [11,12,13]

Sociability aims at the different forms of socialization situations, which arise when individuals engage in reciprocal action through cooperation, competition, and collaboration [14,15,16].

Affectivity refers to individuals’ feelings toward others, encompassing emotions, sentiments, and experiences expressed in social and communicative acts [17,18].

As one prominent author [9] emphasizes, prosocial behaviors align with theories of modeling, highlighting observational learning. All human learning is governed by behaviors guided by observation, modeling, and imitation of others while interacting with environmental and cognitive factors.

There are instances where the behavior and actions of the observing person can be influenced by the positive or negative consequences of the observed model, leaning more towards imitation of that behavior. Prosocial modeling is more pronounced with prosocial behaviors [19].

In today’s society, the daily lives of young people involve continuous exposure and interaction with various networks such as television, video games, and music, all containing a considerable amount of prosocial content [19]. De Vries et al. [20] define prosocial behavior as voluntary assistance toward others, regardless of the intended goal. It is closely related to moral development and human emotions such as help relationships, cooperation, and kindness [21,22].

Therefore, it can be established that human behaviors are related to weighing the cost–benefit balance of actions undertaken. Research studies [23,24,25] on the variables explaining prosocial behavior emphasize the importance of fostering actions of help, unity, patience, and cooperation. Encouraging such actions can prevent and reduce the occurrence and protect individuals against disruptive behaviors. Certain articles provide evidence that mindfulness of a consciously aware person is considered a factor related to internal motivation in the prosocial domain, maintaining an indirect relationship with prosocial behavior through social attention. In other words, this motivation, attention, and behavior aid individuals in translating intrinsic values and motives more effectively [7,8].

There are a series of skills that evidence prosocial behaviors, such as assuming the risks associated with such activities. Adolescents are more inclined to see the opportunity to help rather than the problems that may arise from it [26]. Adolescents may take risks related to negative prosocial aspects of their context (like alcohol consumption, tobacco, deceit...) but, on the other hand, can also acquire positive prosocial aspects (trying new activities, new classes...) [27].

Prosocial behaviors are promoted through the capacity for empathy [28], causing young people to show more concern and pay greater attention to others and their emotions, regardless of their personal characteristics. Adolescents are willing to put them-selves at risk to help others due to the high levels of empathy that motivate them [29]. 

Therefore, they are capable of generating significant positive social changes in society through prosocial and civic behaviors carried out via activities such as volunteering, with the sole aim of contributing to society [30]. This commitment is reflected by considering values of social responsibility and volunteering, which have a connection with prosocial values to benefit others, taking care of themselves and sharing, as well as sharing civic values based on the community’s well-being and collaborating on public issues [31].

Prosocial behaviors in young individuals, where the primary interest is to benefit others, also yield positive outcomes for the individuals themselves, such as high self-esteem and greater academic success [32]. Authors like [33] consider empathy to be one of the predictors of prosocial behavior, motivating individuals to engage in helping behaviors towards others.

Prosocial behaviors increase during adolescence. In this phase, young people gain greater awareness, autonomy increases, and cognitive advancements occur, aiding individuals to engage in prosocial behaviors. This occurs earlier in girls than in boys [34]. These young individuals become small family caregivers, and such activities can yield both positive and negative consequences [35]. Some positive aspects of this activity include fostering self-esteem in these young individuals and building close family bonds [36]. On the flip side, being a caregiver among youths can lead to negative consequences such as reduced social capital or social exclusion, with few opportunities to socialize with peers [37], bullying, physical injuries, feelings of anger, and emotional distress [38]. 

There are volunteering programs where the young caregiver, after spending time with a volunteer adult (considering them as a companion), experiences significant positive benefits. This interaction offers the young caregiver a break from emotional burdens and responsibilities, providing different experiences and contributions determined by the adult’s age [39]. This opportunity for emotional relief through conversations between the young caregiver and the adult companion proves highly beneficial in these programs [39]. The young individuals see this as a chance for respite, obtaining substantial personal benefits [40].

During adolescence, significant physical, cognitive, and relational changes occur that influence social functioning. Firstly, physical maturity enables them to engage in a greater quantity of prosocial actions autonomously [41]. Secondly, adolescents begin to have increased perspective-taking abilities, allowing for greater moral reasoning than in other stages, which in turn contributes to increased prosocial behaviors [42]. Lastly, increased peer interaction and intimate or romantic relationships are closely linked to social behaviors, providing opportunities to increase positive behavior towards others [43].

Due to this latter aspect, human and cultural diversification is considered essential, as it has been on the rise in recent models of current society, leading to greater involvement from individuals to achieve better management and social coexistence [44]. According to the International Organization for Migration, there were 281 million international migrations in 2020, leading to various countries in Asia, Europe, and North America becoming host countries. This diversification resulting from migrations leads to an increase in the number of immigrants, as well as the diversification of their origins, culture, beliefs, and customs, generating socioeconomic, social, personal, and cultural consequences. Hence, there is a need for growth in theories and projects supporting social coexistence [45].

The intercultural approach aims to foster positive interactions among people, promoting mutual respect for diversity and striving for greater equality [46]. It seeks models that fight for greater social inclusion [47]. Giménez [48] considers that this positive interaction will encourage the participation of many individuals, uniting collaboration spaces to build the foundation of interculturalism through collective construction. Diversity is necessary to generate these principles of interculturality, reflecting constructs of coexistence and social cohesion [49]. Therefore, generating an intercultural project seeking to share a common life fosters new identities and ideas for community living [50,51].

Adolescents view voluntary activities as an opportunity to develop their values of social responsibility, reflecting different commitments that aim to help improve society [52]. Volunteer individuals engage in activities in specific organizations without seeking economic benefits, solely aiming to contribute to the community’s benefit [53]. Article 3 of Law 45/2015, of October 14, on Volunteering, considers volunteering as an activity carried out by people following some basic criteria related to solidarity, undertaking this activity freely, without expecting economic or material rewards in return from any entity that offers it.

Chiesa and Stover [53] classify the original motivations for volunteering related to adaptive function, ego-defensive function, cognitive function, and expressive value function established by Katz [54], into six main dimensions termed social and career (strengthening social relationships and personal skills), improvement and protection (reducing negative feelings and growing psychologically), and values and understanding (learning more and expressing oneself in values).

Some authors consider families and friends as the primary socializing agents helping young people develop these prosocial behaviors and values [55]. Young individuals may be involved in various tasks related to caring for others who may suffer from illness [36]. However, there are studies highlighting that girls tend to perform more of this care and support for those in need than boys, especially tasks related to household chores or personal hygiene, which become more important as these young individuals age [56]. Empathetic concern and prosocial behavior are particularly related in girls, establishing a link between that perspective and behavior. These aspects suggest that moral emotions are more connected to prosocial behaviors, playing a significant role in the development of these emotions, especially those related to social understanding, cost–benefit analysis, or greater reasoning [33].

Since the COVID-19 pandemic, one of the prolonged major threats faced by the global population [57], numerous investigations believe it is necessary to address stressors and promote all kinds of collective prosocial responses [58]. These processes not only aid the individual in overcoming stressors but are also crucial for promoting long-term recovery and favoring societies and communities [59].

So, in the context of the past pandemic, numerous voluntary actions have emerged, and concepts such as prosociality are on the rise. People engaging in these activities undertake actions related to helping, caring, comforting, or assisting others in various moments [60]. There are also connections between self-transcendence values and prosociality. Some studies have demonstrated that value orientations, ideological beliefs, and political values are supported by individuals who possess a high value of self-transcendence, thus exhibiting greater prosocial intentions and behaviors [61]. Different types of prosociality emerge, denoted as “bond” prosociality, wherein individuals tend to help those in their immediate surroundings or social network, such as friends or neighbors experiencing vulnerability. However, there are also other forms of prosociality where help extends to vulnerable populations beyond immediate social circles, reaching homeless individuals or those seeking asylum or refuge [62].

Prosociality is directly related to empathic concerns for those in need [63,64]. People with self-transcendent values may perceive the pandemic as a threat to those in need, considering it a threatening facet that drives their assistance more for others’ necessity than personal need [65].

Consequently, post-pandemic, it becomes evident that various values related to others, such as self-transcendence, evoke profound empathic concerns, thereby promoting prosociality to address and mitigate threats faced by vulnerable groups [66]. Studies like [67] have highlighted, after analyzing cross-cultural studies conducted post-pandemic, that cultural and structural aspects have been heightened due to this social issue, resulting in different impacts on individuals, societies, and economies. Therefore, it can be said that there exists cultural and social disparity. It is essential to recognize that individuals, after experiencing significant difficulties that they perceive as a threat, may adopt different core values and prioritize based on their situation during the crisis, leading to varied responses in facing it [68].

Considering the reviewed scientific literature, our objective is to understand how factors such as transcendence, culture, affect, and sociability influence the values of young people, aiming to promote these values in education and enhance prosocial behavior and the hypotheses are as follows: H1: There is a positive association between levels of transcendence and sociability, where individuals with higher levels of transcendence are expected to exhibit greater sociability. H2: Higher levels of transcendence will be positively related to culture, indicating that individuals with stronger transcendental inclinations tend to have a more pronounced cultural orientation. H3: Individuals with beliefs divergent from mainstream religions will exhibit greater cultural diversity, indicating a positive relationship between different beliefs and cultural diversity. H4: There exists a positive correlation between age and levels of affection, suggesting that as individuals age, they tend to display higher levels of affection.

## 2. Materials and Methods

### 2.1. Participants

Sampling was conducted via online access, using a non-probabilistic sampling method by distributing a questionnaire to various entities and collaborating centers. The study collected a total sample of 1702 individuals aged between 18 and 40 years.

In this research, a quantitative study was employed following the empirical–analytical social research method. 

The selected sample had defined characteristics based on the location where the sample was collected, namely the city of Melilla. This small city is situated in North Africa, surrounded by both land and sea borders, converging at the borders of Europe and Morocco. The population in Melilla comprises a high number of civil servants and self-employed workers in small and medium-sized enterprises engaged in local and cross-border trade. Melilla is a multicultural city where the four main cultures (Muslim, Christian, Jewish, and Hindu) coexist in such a confined space, referred to as a melting pot of cultures, emphasizing the need for social coexistence and where a high number of prosocial behaviors exist. Different demographic characteristics such as gender, age, religion, parenthood, Spanish nationality, and participation in voluntary activities were considered in data collection. According to the collected data, the average age of the selected sample was 24.42 years, distributed among 65.9% women and 34.1% men. 57.6% possess undergraduate, university, or postgraduate studies, 24.8% claim to have completed high school studies or equivalent, 16.3% have completed basic or primary studies, and the remaining did not respond to the question. Regarding religious affiliation, 56.5% identified as Christians, 20.2% as Muslims, 0.8% as Jews, 0.2% as Hindus, and 20.8% did not identify with any religion. Notably, in the variable related to the quantity of people involved in voluntary activities, 69.6% claimed to have been volunteers, while 30.4% reported not participating in any voluntary actions. Furthermore, 27.4% were responsible for caring for family members or the elderly, while 70.9% did not have anyone under their care. Regarding parenthood, 78.1% were responsible for children, while 21.9% did not have children.

### 2.2. Instrument

In compliance with data protection and privacy regulations, this study adhered to guidelines where participants consented to the study’s coordinators to process their personal data after being informed about the objectives, purpose, benefits, and the assurance of anonymity. Data were collected during the years 2021 and 2022. 

To design the questionnaire, other instruments from articles such as [69] “Search for values: analysis of axiological content”, and its updated version by González et al. [70], “Analysis and validation of a test to measure values, “and “Questionnaire on values for the promotion of prosocial behaviors” [25] were employed. Using these three instruments, selected items were delimited and evaluated.

The questionnaire was designed using a scoring scale showing the level of agreement with scores ranging from 1 to 3 for each item. To validate the content of the instrument, a three-round Delphi study was conducted [71], allowing for various modifications and suggestions. After the three rounds, the level of agreement was analyzed, resulting in the final questionnaire with a K = 0.87 reliability rate.

Factor validation of the model was established via structural equation modeling (SEM) with AMOS IBM SPSS Statistics 25 program [72]. The sample adequacy assessments conducted to determine the suitability of data for factor analysis included a KMO (Kaiser–Meyer–Olkin) test with a value of 0.879 and a Bartlett sphericity test resulting in 23,224.046 (gl: 741; *p* = 0.000) [25]. These tests collectively suggest that the data is fitting for analysis. Within the SEM, the observed variables were categorized into 4 factors as unobserved exogenous variables. The attained values, assessing the model’s validity, stood at: normalized fit index (NFI = 0.91), incremental fit index (IFI = 0.90), and comparative fit index (CFI = 0.92) [25], all of which were considered satisfactory. Additionally, the root-mean-square error of approximation (RMSEA) reached a value of 0.056, indicating a commendable model fit, thereby confirming its validity according to Knock [73]. The reliability has been calculated using Cronbach’s alpha and has obtained the following: sociability: 0.802; transcendence: 0.927; culture: 0.794; and affections: 0.797.

### 2.3. Procedure

This research adhered to prevailing privacy and data protection laws and standards. Participants willingly provided informed consent for the processing of their personal data, in compliance with the guidelines outlined in Regulation (EU) 2016/679, dated 27 April (GDPR), and Organic Act no. 3/2018, dated 5 December (LOPDGDD). The study was conducted following the agreements of the Helsinki Declaration, subsequently approved by the Research Ethics Committee code ML_22106-3 of Educational Psychology at the University of Granada (201-300 Academic Ranking of World Universities, Shanghai, 2018).

The questionnaire used for data collection was also approved by the academic committee responsible for the Faculty of Education at the University of Granada, conducted through the online platform Google Forms. Statistical analysis was conducted using IBM SPSS Statistics 25 software. A multiple linear regression analysis (employing the enter method) was utilized to organize and categorize values into dimensions. In this analysis, each individual was considered the dependent variable, while different dimensions representing prosocial behavior were employed as predictor variables [25].

The identification of factor groupings resulted in four factors: Sociability—involving social interaction and interpersonal relations. Transcendence—encompassing relationships perceived to extend beyond natural boundaries. Culture—comprising lifestyle considerations, artistic expressions, and social group affiliations. Affection—focusing on emotional inclinations and mood tendencies.

### 2.4. Statistical Analysis of the Data

**Figure A1:**
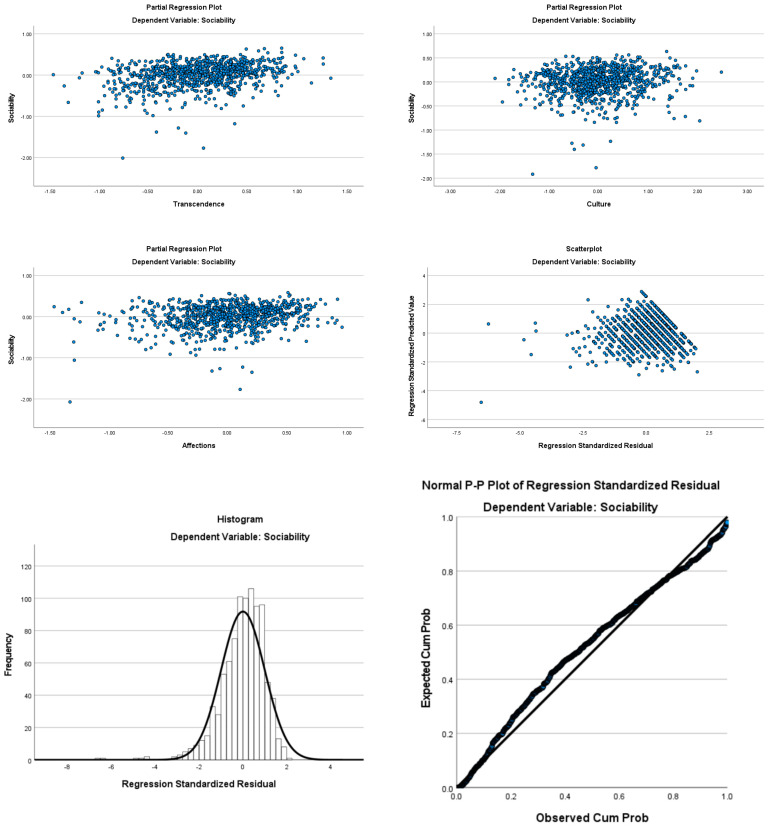
Partial regression plots of the criterion variable sociability with the predictor variables transcendence, culture, and affections; scatterplot; and assumption of normality (histogram and normal probability plot) of the criterion variable sociability.

The multivariate analysis technique of stepwise multiple linear regression (MLR) was applied to find out the predictive value of four assumed models (Table 1), whose input variables in each of the proposed models can be found in Figure A1 (Appendix A).

The analyses carried out revealed the assumptions of all the models, with the exception of homoscedasticity, which was tested with Leven’s test (*p* > 0.05).

## 3. Results

**Figure A2:**
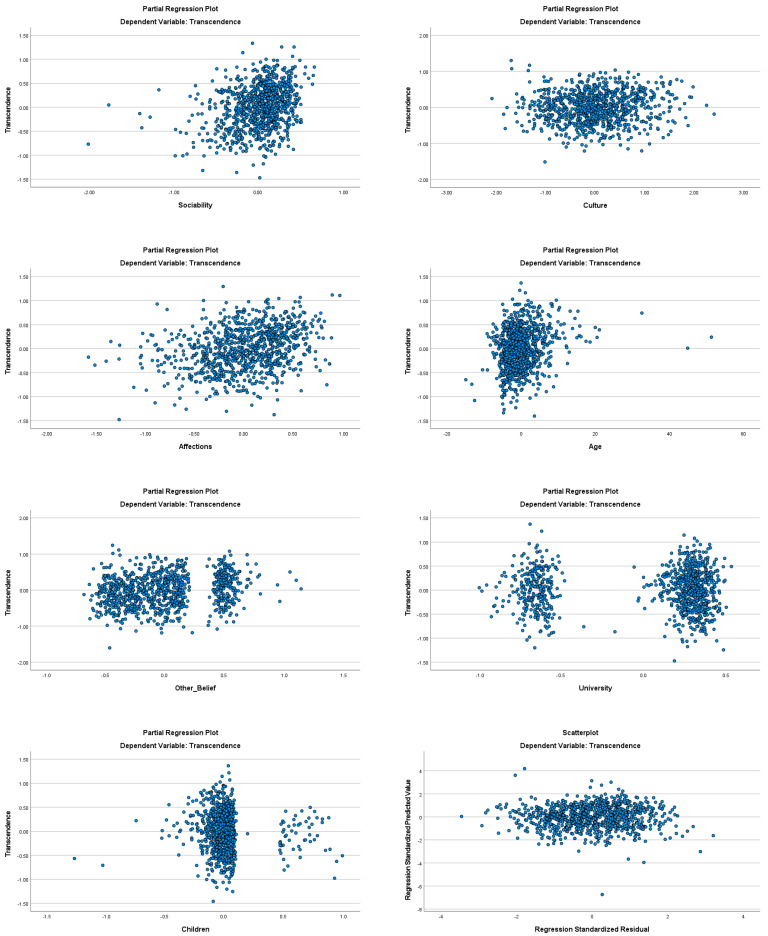

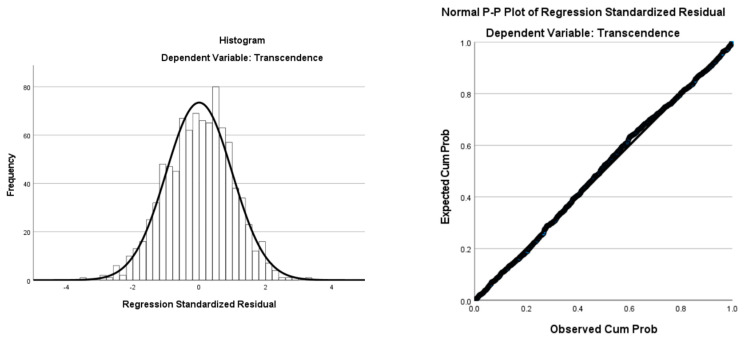
Partial regression plots of the criterion variable transcendence with the predictor variables sociability, culture, affections, age, other belief, university, and children; scatterplot; and normality assumption (histogram and normal probability plot) of the criterion variable transcendence.

**Figure A3:**
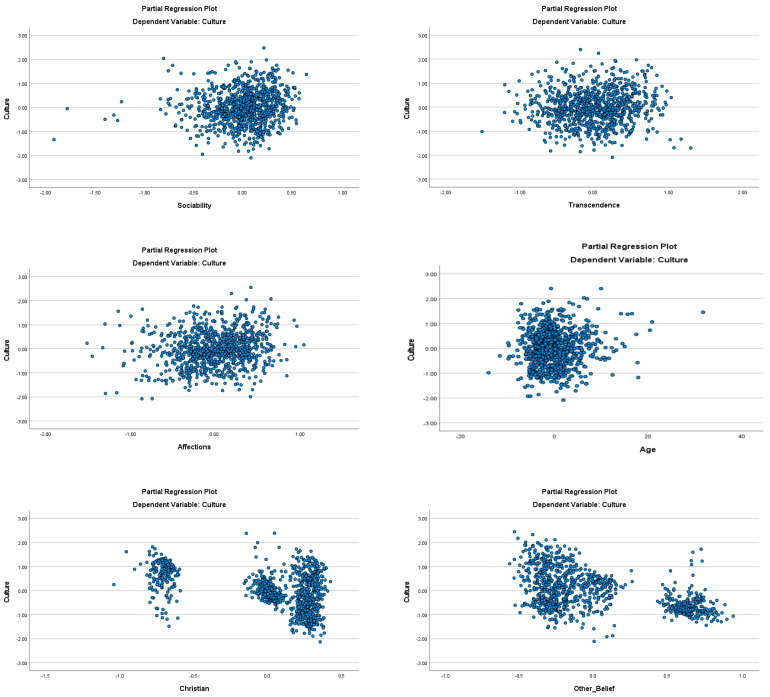

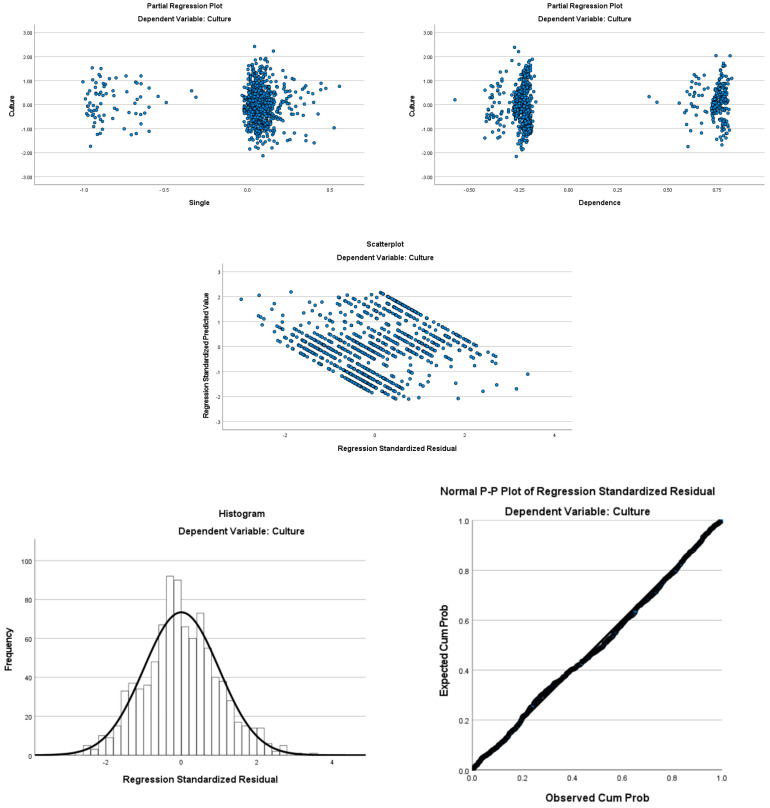
Partial regression plots of the criterion variable culture with the predictor variables sociability, transcendence, and affections, age, Christian, other belief, single, and dependence; scatter plot; and assumption of normality (histogram and normal probability plot) of the criterion variable culture.

**Figure A4:**
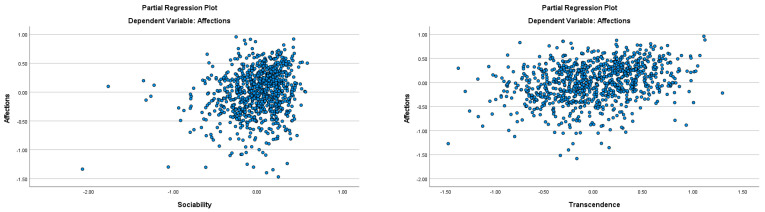

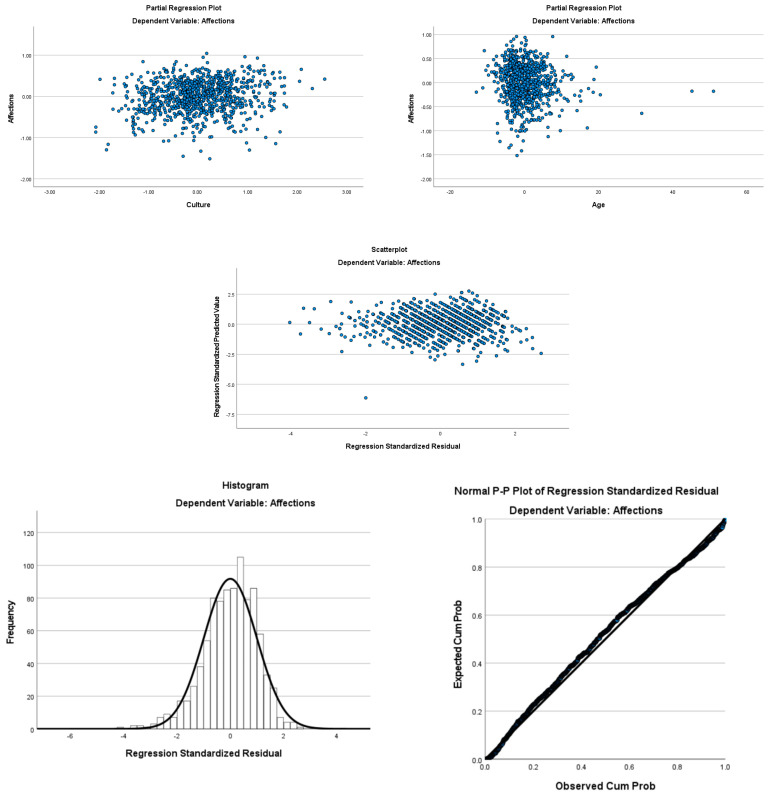
Partial regression plots of the criterion variable affections with the predictor variables sociability, transcendence, culture, and age; scatter plot; and assumption of normality (histogram and normal probability plot) of the criterion variable sociability.

The MLR indicates that the assumptions of the four models are met, hence the validity of the proposed models [74]. Following Vilá [75], they have been verified by checking in each model that the assumption of linearity (partial scatter plots in Figure A1, Figure A2, Figure A3 and Figure A4), independence of errors (Durbin–Watson value between 1.5 and 2.5, in Table A1, Table A2, Table A3 and Table A4), normality (Figure A1, Figure A2, Figure A3 and Figure A4) (see Appendix A), homoscedasticity (*p* > 0.05), and non-collinearity (tolerance > 0.10, and variance inflation factor < 10; Table 2, Table 3, Table 4 and Table 5).

### 3.1. Predictive Model of Sociability

The MLR analysis suggested 3 models, with the last one offering the highest explanatory power (Table A1, Appendix A). The goodness of fit of the model is adequate, with sociability being explained by 22.3% of the variance contributed by three variables out of the twelve introduced in the model (transcendence, culture, and affections). Thus, the variables age, Christian, Islamic, Jew, other belief, single, children, university, and dependence are excluded.

Table 2 shows the values of interest for the selected predictive model. The t-value is associated with a probability of error of less than 0.001 for the three variables included in the model. Also, the standardized coefficients indicate which variables have a higher explanatory weight in the model. Transcendence is the strongest predictor of sociability (β = 0.311). To a lesser extent, it is followed by affections (β = 0.215) and culture (β = 0.110).

Therefore, a person’s sociability increases the higher his or her transcendence, affections, and culture. The predictive equation representing this relationship is as follows:

Sociability = 2.480 + 0.311 Transcendence + 0.215 Affections + 0.110 Culture

where sociability is the score a person would have on his or her sociability; 2.480 the constant of the equation; transcendence, affections, and culture the score obtained by the person on these constructs.

### 3.2. Predictive Model of Transcendence

Seven models emerged, model 7 being the one with the highest explanatory power (Table A2, Appendix A). The goodness of fit of the model is adequate, with 31.1% of the variance in Transcendence being explained by seven of the twelve variables introduced in the model: sociability, affections, age, other belief, culture, children, and university. Therefore, Christian, Islamic, Jew, single, and dependence are excluded.

In relation to the selected predictive model, Table 3 shows that the t-value is associated with a probability of error of less than 0.05 for the seven variables included in the model. Likewise, the standardized coefficients indicate which variables present a greater explanatory weight in the model. Affections is the strongest predictor of a person’s transcendence (β = 0.282). It is followed to a lesser extent by their level of sociability (β = 0.278), their age (β = 0.248), other belief (β = 0.227), their degree of culture (β = 0.087), whether or not they have children (β = −0.077), and whether or not they have a university education (β = 0.060).

Therefore, a person will have greater transcendence the higher the degree of affections, sociability, and culture present, as well as the higher the age. It also influences the older the person is, the more he/she is, the more he/she identifies with other beliefs or at least does not specify his/her spiritual ideology (other belief), the more he/she does not have children, and the more he/she has a university education. However, it is higher in the case of not having children. Specifically, to calculate a person’s transcendence, its predictive equation is as follows:

Transcendence = −0.273 + 0.282 Affections + 0.278 Sociability + 0.248 Age + 0.227 Other Belief + 0.087 Culture − 0.077 Children + 0.060 University

where transcendence is the score that a person would have on their level of transcendence; −0.273 the constant of the equation; affections, sociability, and culture the score obtained in these constructs; their age as a predictive factor; other beliefs without express identification of the same; children corresponds to the situation of having or not having children, which having a negative value specifies the lack of them as a predictive factor; university indicates whether or not they have university studies, with having such studies standing out as a predictive factor.

### 3.3. Predictive Model of Culture

A total of eight models were proposed based on the MLR analysis, and the last one was selected as it offered the greatest explanatory capacity (Table A3, Appendix A). The goodness of fit of this model can be considered adequate, with 49.7% of the variance in culture being explained by eight of the twelve variables introduced in the model (other belief, Christian, affections, age, sociability, dependence, transcendence, and single). Thus, the variables Islamic, Jew, children, and university are excluded from the model.

In relation to the selected predictive model, Table 4 shows that the t-value is associated with an error probability of less than 0.05 in the eight variables included in the model (other belief, Christian, affections, sociability, dependence, single, age, and transcendence). Taking into account the standardized coefficients, to indicate which variables have a greater explanatory weight in the model, it is found that having other belief is the strongest predictor of the person’s culture (β = −0.832). To a lesser extent, it is followed by being Christian or not (β = −0.472), their level of affections (β = 0.128), their level of sociability (β = 0.076), being in charge of a dependent person (β = 0.065), being single or not (β = −0.063), their age (β= 0.062), and their degree of transcendence (β = 0.061).

Therefore, a person will have a higher culture the higher his or her tendency to have other belief (not identifying with any religious ideology), higher level of affections and sociability, having a dependence, not being single, higher age, and higher degree of transcendence. The predictive equation for a person’s culture is as follows:

Culture = 1.066 − 0.832 Other Belief − 0.472 Christian + 0.128 Affections + 0.076 Sociability + 0.065 Dependence − 0.063 Single + 0.062 Age + 0.061 Transcendence

where culture is the score a person would have on their culture; 1.066 is the constant in the equation; other belief is their belief related to religion, other than Christianity, Islam, and Judaism; Christian is being Christian; affections, sociability, and transcendence, their respective scores on those constructs; dependence indicates whether or not they are a dependent; single indicates whether or not they are single; and age is their age.

### 3.4. Predictive Model of Affections

The MLR analysis suggested four models, with model 4 offering the highest explanatory power (Table A4 in Appendix A). Its goodness of fit is considered adequate, with 24.1% of the variance in a person’s affections being explained by four of the twelve variables entered in the model (transcendence, sociability, culture, and age). Christian, Islamic, Jew, other belief, single, children, university, and dependence are excluded.

In relation to the selected predictive model, Table 5 shows that the t-value is associated with an error probability of less than 0.05 for the four variables included in the model. Likewise, the standardized coefficients indicate which variables have a greater explanatory weight in the model. Specifically, the degree of affections is the strongest predictor of its degree of significance (β = 0.300). To a lesser extent, it is followed by their degree of sociability (β = 0.210), their degree of culture (β = 0.200), and their age (β = −0.114). Therefore, the more affections a person has, the more transcendence, sociability, and culture they have, but the less age they have.

The predictive equation arrived at with the RLM analysis to predict a person’s level of affections is as follows:

Affections = 1.321 + 0.300 Transcendence + 0.210 Sociability + 0.200 Culture − 0.114 Age

where we have a person’s degree of affections, the constant of the equation (1.321), the person’s degree of transcendence, his or her degree of sociability, his or her cultural level, and age.

## 4. Discussion

The results obtained the following conclusions in this study group related to the values that promote prosocial behaviors. Regarding the value of sociability, the results show that it is favored and increased by the value of transcendence, as it is the strongest value, followed by affectionate individuals and those with cultural values. Therefore, sociability is greatly influenced by individuals with higher spiritual beliefs, as these individuals tend to foster prosocial behaviors. There exists a relationship between different values of self-transcendence, value orientations, ideological beliefs, and political values, all of which contribute to a higher intention and prosocial behaviors [20,21,22,23,25].

Regarding the value related to transcendence, the individuals scoring the highest are affectionate persons, as they obtain a higher value and thus exert more influence. This is due to the association between emotions and focal strengths such as appreciation of beauty, hope, spirituality, among others. This association provides an opportunity to reinforce and inspire prosocial behaviors by generating positive emotions [11]. Following affectionate individuals, a person’s level of sociability also influences transcendence, with the last aspect being the individual’s age [19]. Age can also be a factor affecting this, as different research highlights that young people engage in support activities for those in need, and as people grow older, this involvement gains more significance [12,13]. Furthermore, other influencing factors for transcendence include different beliefs, the cultural degree of individuals, and lastly, whether they have children or hold university degrees.

The factor most related to culture is having different beliefs; individuals with beliefs different from the normalized ones contribute the most to cultural aspects. This phenomenon occurs because various cultures coexist in multicultural spaces, allowing the development of languages and diverse beliefs. Multiculturalism represents an opportunity for acceptance and positive coexistence due to this diversity [13,50]. Moreover, individuals who consider themselves non-Christian obtain higher scores, thus elevating the cultural level. Culture is likened to nature, indicating a shift from Christianization towards inculturation, associating culture more with the term “nature” than with the path of faith [10,13]. Additionally, variables such as an individual’s level of affection, sociability, responsibility for dependents, marital status, and finally, the degree of transcendence, affect culture, with spirituality being the least influential factor.

Regarding an individual’s affection, the constant that exerts the most influence is the person’s level of transcendence, once again highlighting an individual’s spirituality as the primary factor. This is followed by individuals who are more sociable and possess a higher level of culture. Lastly, age affects affection, with younger individuals needing to display more affection. This can be attributed, in part, to the fact that young people nowadays use social networks to generate new social exchanges with different values and emotions. This emotional situation can significantly influence others and impact the non-virtual world. Positive messages on social media can evoke negative emotions through methods like upward social comparison, but they can also work positively by emotional contagion [20,25].

## 5. Conclusions

The results demonstrate that the four main values in this study—sociability, transcendence, culture, and affection—need improvement and further work through increased social interaction across different environments and communities, via formal or informal voluntary activities. Therefore, it is paramount to work on the development of prosocial behavior through voluntary assistance to others. Volunteering is closely linked to moral development and human emotions such as relationships of help, cooperation, and kindness. From a psychological perspective, prosocial behavior and altruism are grounded in experience. Hence, at different stages of development, individuals undergo a series of social experiences through social connections and communication, leading to the learning of social behavior. Culture is the factor least affecting sociability and transcendence, likely due to the challenge of coexistence caused by cultural diversity, where some cultures develop more than others. Sociability and transcendence values are affected by culture, hence the need to develop socio-cultural models adapted to the needs that promote social coexistence among different cultures while respecting everyone’s spirituality within the same city. This will involve models of coexistence that foster these social, transcendent, and cultural values.

Regarding culture, there is a need to improve individuals’ level of transcendence, as spirituality obtains the lowest score when associated with the cultural factor, being the least influential aspect. Therefore, joint voluntary activities reflecting both cultural and spiritual characteristics could be carried out. Culture and age are the aspects that need enhancement concerning the variable related to affection. Thus, leveraging young people and digital platforms to promote cultural gatherings fostering connections between different cultures can enhance these affective behaviors. These platforms could be utilized to spread the word about upcoming activities, coupled with the necessity of creating cultural and socio-affective programs that provide young people with opportunities to learn about different heritages, histories, and cultures.

Creating communities capable of having this cultural and spiritual vision will lead to viable and sustainable coexistence communities, promoting an advantageous situation for enhancing social participation.

Limitations: The study incorporates a specific set of variables (transcendence, affections, culture, sociability) which might not encompass all aspects influencing the identified constructs. Additional variables might enhance the predictive power of the models. The exclusion of certain variables (e.g., Islamic, Jew, Christian) may limit the comprehensive understanding of the studied phenomena, potentially overlooking valuable correlations. Model’s predictive power: Although the proposed models demonstrate explanatory power, the variance explained in some cases remains modest.

Future directions and proposals:

Incorporation of additional variables: Expand the range of variables considered for the models to include a more comprehensive array of factors that could influence the identified constructs, thereby potentially improving the predictive capability of the models.

Cultural and socio-affective programs: Develop and implement programs that leverage the findings, fostering socio-cultural understanding, intercultural connections, and socio-affective development, particularly targeting younger individuals through digital platforms and community-based initiatives.

Community-based interventions: Focus on creating communities that embrace cultural and spiritual diversity, aiming to enhance social participation and foster a conducive environment for sustainable coexistence and positive social behaviors.

Longitudinal studies: Engage in longitudinal studies to capture changes and evolution in these constructs over time, enabling a deeper understanding of the dynamics and potential causal relationships among them.

Cross-cultural studies: Explore comparative studies across diverse cultural contexts to discern how cultural nuances might impact the identified values and their interrelations.

These future directions and proposals aim to address the limitations observed in the study while paving the way for more comprehensive, robust, and applicable models in understanding and promoting prosocial behaviors and cultural dynamics.

## Figures and Tables

**Table 1:** Predictive value of four assumed models.

Variables introducidas en los modelos	Model 1	Dependent variable: sociability.
		Predictor variables: transcendence, culture, affections, Christian ^1^, Islamic ^2^, Jew ^3^, other belief ^4^, single ^5^, children ^6^, university ^7^, dependence ^8^.
	Model 2	Dependent variable: transcendence.
		Predictor variables: sociability, culture, affections, Christian ^1^, Islamic ^2^, Jew ^3^, other belief ^4^, single ^5^, children ^6^, university ^7^, dependence ^8^.
	Model 3	Dependent variable: culture.
		Predictor variables: sociability, transcendence, affections, Christian ^1^, Islamic ^2^, Jew ^3^, other belief ^4^, single ^5^, children ^6^, university ^7^, dependence ^8^.
	Model 4	Dependent variable: affections
		Predictor variables: sociability, transcendence, culture, Christian ^1^, Islamic ^2^, Jew ^3^, other belief ^4^, single ^5^, children ^6^, university ^7^, dependence ^8^.
Note: ^(1)^ Professes the Christian religion; ^(2)^ Professes the Islamic religion; ^(3)^ Professes the Jewish religion; ^(4)^ Professes another religion or belief; ^(5)^ Is or is not single; ^(6)^ Has or does not have children; ^(7)^ Has or does not have a university education; ^(8)^ Is or is not in charge of a dependent.		

**Table 2:** Coefficients in the multiple linear regression model equation for predicting a person’s Sociability.

Model ^a^		Non-Standardized Coefficients		Standardized Coefficients	*t*	*p*	Collinearity Statistics	
		Β	Error Stand.	Β			Tolerance	VIF
3	(Constant)	2.480	0.073		33.989	<0.001		
	Transcendence	0.196	0.020	0.311	9.890	<0.001	0.855	1.169
	Affections	0.158	0.024	0.215	6.624	<0.001	0.805	1.243
	Culture	0.035	0.010	0.110	3.658	<0.001	0.930	1.075

^a^ Dependent variable: sociability of people.

**Table 3:** Coefficients in the equation of the multiple linear regression model for predicting a person’s transcendence.

Model ^a^		Non-Standardized Coefficients		Standardized Coefficients	*t*	*p*	Collinearity Statistics	
		Β	Error Stand.	Β			Tolerance	VIF
7	(Constant)	−0.273	0.175		−1.565	0.118		
	Sociability	0.442	0.047	0.278	9.349	<0.001	0.847	1.181
	Affections	0.329	0.035	0.282	9.343	<0.001	0.826	1.210
	Age	0.019	0.003	0.248	7.313	<0.001	0.653	1.531
	Other Belief	0.268	0.039	0.227	6.937	<0.001	0.699	1.431
	Culture	0.044	0.017	0.087	2.543	0.011	0.645	1.551
	Children	−0.162	0.072	−0.077	−2.270	0.023	0.660	1.516
	University	0.028	0.013	0.060	2.185	0.029	0.990	1.010

^a^ Dependent variable: Transcendence.

**Table 4:** Coefficients in the equation of the multiple linear regression model for predicting a person’s culture.

Model ^a^		Non-Standardized Coefficients		Standardized Coefficients	*t*	*p*	Collinearity Statistics	
		Β	Error Stand.	Β			Β	Error Stand.
8	(Constant)	1.066	0.338		3.150	0.002		
	Other Belief	−1.924	0.072	−0.832	−26.726	<0.001	0.566	1.767
	Christian	−0.952	0.063	−0.472	−15.229	<0.001	0.571	1.750
	Affections	0.293	0.061	0.128	4.793	<0.001	0.772	1.295
	Age	0.010	0.005	0.062	2.012	0.045	0.573	1.744
	Sociability	0.236	0.083	0.076	2.859	0.004	0.781	1.281
	Dependence	0.151	0.055	0.065	2.766	0.006	0.996	1.004
	Transcendence	0.120	0.055	0.061	2.176	0.030	0.692	1.445
	Single	−0.235	0.111	−0.063	−2.118	0.034	0.614	1.630

^a^ Dependent Variable: Culture.

**Table 5:** Coefficients in the equation of the multiple linear regression model to predict affections of a person.

Model ^a^		Non-Standardized Coefficients		Standardized Coefficients	*t*	*p*	Collinearity Statistics	
		Β	Error Stand.	Β			Β	Error Stand.
4	(Constant)	1.321	0.146		9.044	<0.001		
	Transcendence	0.256	0.028	0.300	9.304	<0.001	0.798	1.253
	Sociability	0.286	0.043	0.210	6.573	<0.001	0.811	1.233
	Culture	0.087	0.013	0.200	6.808	<0.001	0.954	1.048
	Age	−0.008	0.002	−0.114	−3.844	<0.001	0.943	1.060

^a^ Dependent variable: Affections.

## Data Availability

Data are available in principal research mcolmos@ugr.es.

## Appendix A

**Table A1:** Stepwise multiple linear regression model to predict a person’s sociability.

Model ^d^	*R*	*R* ^2^	*R*^2^ Corrected	Standard Error of the Estimate	g.l.	*F*	*p* de *F*	Durbin–Watson
1	0.405 ^a^	0.164	0.163	0.29476	1	179.603	<0.001	
2	0.463 ^b^	0.215	0.213	0.28583	2	125.098	<0.001	
3	0.475 ^c^	0.226	0.223	0.28391	3	88.985	<0.001	1.893

^a^ Predictor variables: (Constant), transcendence; ^b^ Predictor variables: (Constant), transcendence, affections; ^c^ Predictor variables: (Constant), transcendence, affections, culture; ^d^ Dependent Variable: sociability.

**Table A2:** Stepwise multiple linear regression model to predict a person’s transcendence.

Model ^h^	*R*	*R* ^2^	*R*^2^ Corrected	Standard Error of the Estimate	g.l. Total	*F*	*p* de *F*	Durbin–Watson
1	0.405 ^a^	0.164	0.163	0.46834	918	179.603	<0.001	
2	0.476 ^b^	0.227	0.225	0.45063	918	134.233	<0.001	
3	0.519 ^c^	0.269	0.267	0.43831	918	112.334	<0.001	
4	0.552 ^d^	0.304	0.301	0.42784	918	100.008	<0.001	
5	0.556 ^e^	0.309	0.305	0.42665	918	81.670	<0.001	
6	0.559 ^f^	0.313	0.308	0.42574	918	69.166	<0.001	
7	0.562 ^g^	0.316	0.311	0.42487	918	60.212	<0.001	1.873

^a^ Predictor variables: (Constant), sociability; ^b^ Predictor variables: (Constant), sociability, affections; ^c^ Predictor variables: (Constant), sociability, affections, age; ^d^ Predictor variables: (Constant), sociability, affections, age, other belief; ^e^ Predictor variables: (Constant), sociability, affections, age, other belief, culture; ^f^ Predictor variables: (Constant), sociability, affections, age, other belief, culture, children; ^g^ Predictor variables: (Constant), sociability, affections, age, other belief, culture, children, university; ^h^ Dependent Variable: transcendence.

**Table A3:** Stepwise multiple linear regression model for predicting a person’s culture.

Model ^i^	*R*	*R* ^2^	*R*^2^ Corrected	Standard Error of the Estimate	g.l. Total	*F*	*p* de *F*	Durbin–Watson
1	0.540 ^a^	0.292	0.291	0.84626	918	378.307	<0.001	
2	0.661 ^b^	0.437	0.436	0.75482	918	356.082	<0.001	
3	0.685 ^c^	0.469	0.467	0.73365	918	269.484	<0.001	
4	0.696 ^d^	0.485	0.482	0.72325	918	214.849	<0.001	
5	0.701 ^e^	0.492	0.489	0.71858	918	176.708	<0.001	
6	0.704 ^f^	0.496	0.493	0.71590	918	149.664	<0.001	
7	0.706 ^g^	0.498	0.495	0.71462	918	129.352	<0.001	
8	0.708 ^h^	0.501	0.497	0.71326	918	114.177	<0.001	1.727

^a^ Predictor variables: (Constant), other belief; ^b^ Predictor variables: (Constant), other belief, Christian; ^c^ Predictor variables: (Constant), other belief, Christian, affections; ^d^ Predictor variables: (Constant), other belief, Christian, affections, age; ^e^ Predictor variables: (Constant), other belief, Christian, affections, age, sociability; ^f^ Predictor variables: (Constant), other belief, Christian, affections, age, sociability, dependence; ^g^ Predictor variables: (Constant), other belief, Christian, affections, age, sociability, dependence, transcendence; ^h^ Predictor variables: (Constant), other belief, Christian, affections, age, sociability, dependence, transcendence, single; ^i^ Dependent variable: culture.

**Table A4:** Stepwise multiple linear regression model for predicting a person’s affections.

Model ^e^	*R*	*R* ^2^	*R*^2^ Corrected	Standard Error of the Estimate	g.l. Total	*F*	*p* de *F*	Durbin–Watson
1	0.380 ^a^	0.145	0.144	0.40560	1	155.078	<0.001	
2	0.443 ^b^	0.197	0.195	0.39331	2	112.057	<0.001	
3	0.482 ^c^	0.232	0.229	0.38475	3	92.144	<0.001	
4	0.494 ^d^	0.244	0.241	0.38189	4	73.842	<0.001	1.997

^a^ Predictor variables: (Constant), transcendence; ^b^ Predictor variables: (Constant), transcendence, sociability; ^c^ Predictor variables: (Constant), transcendence, sociability, culture; ^d^ Predictor variables: (Constant), transcendence, sociability, culture, age; ^e^ Dependent variable: affections.
